# The learning curve of endoscopic thyroid surgery for papillary thyroid microcarcinoma: CUSUM analysis of a single surgeon’s experience

**DOI:** 10.1007/s00464-018-6410-y

**Published:** 2018-09-27

**Authors:** Jian Yu, Shangrui Rao, Zhe Lin, Zhongliang Pan, Xiangjian Zheng, Zhonglin Wang

**Affiliations:** Department of General Surgery, Theorem Clinical College of Wenzhou Medical University, Wenzhou Central Hospital, Wenzhou, 32 Dajian Lane, Wenzhou, 325000 Zhejiang China

**Keywords:** Endoscopic thyroid surgery, CUSUM, Learning curve, Papillary thyroid microcarcinoma, Central neck dissection

## Abstract

**Background:**

With the development of surgical technics, endoscopic thyroid surgery has been gradually accepted and utilized in thyroid disease treatment, including thyroid carcinoma. This study aimed to evaluate the learning curve for endoscopic hemithyroidectomy (EHT) with ipsilateral central neck dissection (CND) and investigate how many cases must be performed before a surgeon becomes competent and proficient in this approach.

**Methods:**

Ninety-nine consecutive patients who underwent EHT with ipsilateral CND for papillary thyroid microcarcinoma by a single surgeon between June 2015 and October 2017 were analyzed. Multidimensional cumulative summation (CUSUM) analysis was performed to evaluate the learning curve.

**Results:**

The CUSUM graph showed the learning curve ascended in the first 31 cases and declined in the following cases. The number of lymph nodes removed in phase 2 (the following 68 cases) was significantly more than that in phase 1 (the first 31 cases) (5.06 ± 1.44 vs. 4.19 ± 1.51, *P* = 0.001). The operation time in phase 2 was shorter than that in phase 1 (123.38 ± 12.71 min vs. 132.90 ± 13.95 min, *P* = 0.008) and the rate of accidental removal of parathyroid gland decreased from 35.5% in phase 1 to 16.2% in phase 2 (*P* = 0.040). There was a declining trend but no significant difference in the rate of postoperative complications (9.7% in phase 2 vs. 4.4% in phase 1, *P* = 0.309).

**Conclusion:**

EHT with ipsilateral CND performed by surgeons was mastered after 31 cases, and the safety and feasibility of this endoscopic approach can also be demonstrated.

Papillary thyroid microcarcinoma (PTMC) deserves attention because of its increasing frequency among patients with thyroid papillary carcinoma in clinical practice and its implications for patient management involving young adults [1]. Conventional thyroid surgery has been a safe and efficient procedure for more than 100 years. However, since the first endoscopic thyroidectomy (ET) was reported in 1997 [2], various endoscopic approaches have been applied to thyroid surgery [3, 4]. Their applications were not only for benign thyroid tumors but also for the malignant ones. Previous research has indicated that endoscopic thyroid surgery is an effective alternative for selected patients with PTMC when compared with conventional open thyroid surgery [5]. But as a new surgical technique, endoscopic thyroid surgery requires skilled surgeons and a long learning curve. Several studies have investigated the learning curve for endoscopic thyroid surgery, but they were mostly based on operation time [6–9], with few studies specializing in PTMC. In our study, patients with PTMC underwent endoscopic hemithyroidectomy (EHT) with ipsilateral central neck dissection (CND). To accurately and comprehensively evaluate the learning curve for EHT with ipsilateral CND, the multidimensional cumulative summation (CUSUM) analysis, which can enable investigators to visualize the data for trends was performed based on chronological cases. The aim of present study was to investigate how many cases must be performed before a surgeon becomes competent and proficient in performing EHT with ipsilateral CND.

## Materials and methods

### Patients

Between June 2015 and October 2017, patients diagnosed as PTMC were included in a prospective observational study at the general surgical department of Wenzhou Central Hospital. These patients underwent endoscopic thyroid surgery by a single surgeon who had been engaged in clinical work of general surgery for about 10 years and had experience of at least 300 cases in conventional thyroid surgery for 3 years and at least 60 cases in endoscopic thyroidectomy for 1 year but never performed CND in endoscopic surgery. The inclusion criteria included (1) papillary thyroid carcinoma was diagnosed by fine needle aspiration (FNA) preoperative or frozen section examination intraoperative; (2) the diameter of primary tumor was < 10 mm as indicated in imaging examination; (3) there was no evidence indicating lymph node (LN) metastasis, extrathyroidal invasion, and distant metastasis; (4) endoscopic operation was consented by the patients. The excluded criteria was (1) the endoscopic procedure was not tolerated; (2) preoperative thyroid function was abnormal; (3) the patients have previous history of radiotherapy or operation at the neck; (4) chronic thyroid inflammation was indicated in the preoperative imaging examination or FNA; and (5) two or more malignant tumors were distributed in bilateral thyroid gland. The surgical procedure was recommended to all patients, and information about potential complications was given. All patients gave written informed consent for participation in the study and the study was registered with Chinese Clinical Trial Registry (Trial Registry Number: ChiCTR-OOC-17011969).

### Operation procedure

Routine preparation consisted of general anesthesia and endotracheal intubation. The patient was placed in the supine position with the neck extended slightly. The whole endoscopic surgical procedure was described detailedly in the following. A 10-mm longitudinal incision was made at the interior edge of the right breast areolae; two other 5-mm incisions at the lateral edge of the breast areolae were made (two incisions, respectively, at both sides of the middle point of breast areolae and sternoclavicular joint in male patients). Epinephrine diluted in 0.9%NaCl solution (1:500,000) was injected into the working area under the platysma in the neck and subcutaneously in the anterior chest. A 10-mm trocar and two 5-mm trocars were then punctured into the subcutaneous space. A 10-mm 30° laparoscope (Olympus, Tokyo, Japan) was then introduced through the 10-mm trocar. An ultrasound scalpel (Johnson & Johnson Medical, Cincinnati, OH) and an assistant clamp were then introduced through the 5-mm trocars. There was room for manipulation from the thyroid cartilage superiorly to 4 cm below the suprasternal fossa inferiorly and laterally from just beyond the medial border of the sternocleidomastoid muscle under euthyphoria, which was established using the ultrasound scalpel and maintained using low-pressure CO_2_ insufflation at 5–6 mmHg. Incision of the linea alba cervicalis and dissection of the thyroid were done using the harmonic scalpel. The middle thyroid vein and inferior thyroid vessels were identified and divided. After the isthmus was divided, the lower lobe of the thyroid gland was drawn upward and bluntly dissected. The parathyroid gland (PTG) was identified and carefully preserved, with intact blood supply. The superior thyroid vessels were identified and dissected close to the thyroid gland to avoid injuring the superior laryngeal nerve. The recurrent laryngeal nerve (RLN) was traced and dissected with great care during the complete lobectomy procedure. The resected specimen was placed into a removal bag, extracted through the subcutaneous cavity of the 10-mm trocar to the skin incision and sent for frozen histological examination. Ipsilateral CND was made when PTMC was confirmed by the frozen histological examination after about a 30-min wait. The central LN was defined as nodes bordered superiorly by the hyoid bone, inferiorly by the innominate (brachiocephalic) artery, and laterally on each side by the carotid sheaths. The RLN was traced and dissected with great care during ipsilateral CND. This specimen was extracted with the same method and sent for postoperative pathological examination. After adequate irrigation, a single drain was placed in the cavity through the skin incision. Suture of the strap muscles and skin incision was closed with absorbable sutures. The operation time was measured from skin incision to wound closure except for the time waiting for the frozen histological examination.

### CUSUM analysis

CUSUM analysis is a statistical technique applied to surgical procedures for the quantitative estimation of the learning curve [10]. The standard CUSUM analysis shows the cumulative differences between the observed data and the target value. Some studies used CUSUM analysis based only on the single dimension, but in the present study, we performed a multidimensional analysis of learning curve as described below [11].

To perform multidimensional CUSUM analysis, we designated the number of removed LNs, operation time, postoperative complications, and accidental removal of PTG as the assessment indicators of surgical competence. These four assessment indicators were, respectively, set as quantized value *a*_1_, *a*_2_, *a*_3_, and *a*_4_ for each case. The quantized value of assessment indicator was defined as *a* = *X*_i_ − *X*_0_, where *X*_*i*_ was an individual attempt and *X*_0_ was the reference or target value for the procedure, with *X*_i_ = 1 for attempt below standard (the number of removed LNs or operation time) or failure (postoperative complication or accidental removal of PTG) and *X*_i_ = 0 for attempt up to standard (the number of removed LNs or operation time) or success (no postoperative complication or no accidental removal of PTG). In accordance with other surgeons’ experience in previous studies and our experience in the present study, the target rates for the number of removed LNs and the operation time were, respectively, set at 5 and 125 min [1, 9, 12, 13]. The rates of attempt up to standard in the present study were, respectively, 42% and 44%. The target occurrence rates for postoperative complication and accidental removal of PTG were set at 5% and 20% [9, 12, 14], respectively. The *X*_0_ for these four assessment indicators were, respectively, 0.42, 0.44, 0.05, and 0.2. So, the quantized value of surgical competence for each case was defined as *S* = *a*_1_ + *a*_2_ + *a*_3_ + *a*_4_. After each case, scores were sequentially added and then plotted graphically by the equation: CUSUM = ∑*S*_*i*_.

It was based on CUSUM graph that fit a polynomial curve was used to depict the learning curve. A positive slope implied that the target was not achieved, and a negative slope suggested that the target had been exceeded. The transition point of slope from positive to negative reflected the mastery of the surgical procedure.

### Statistical analysis

SPSS v. 19.0 for Windows analyzed all data. The continuous variable was expressed as the mean and standard deviation (SD). Comparisons among groups were performed using the Chi-square test for discrete variables and *t* test for continuous variables. *P* values < 0.05 (two-tailed) were considered statistically significant.

## Results

Ninety-nine consecutive patients diagnosed with PTMC who underwent EHT with ipsilateral CND were included in the present study. The mean age of these patients was 36.99 years old. The mean size of primary thyroid tumors was 6.84 mm while the mean operation time was 126.36 min. The total postoperative complications rate was 6% (6 of 99). The important characteristics and operative outcomes are shown in Table 1.

**Table 1:** Patient characteristics and operative outcomes

	Total (*n* = 99)	Phase 1 (*n* = 31)	Phase 2 (*n* = 68)	*P* value
Age (years)	37.65 ± 7.59	37.58 ± 9.76	37.67 ± 6.46	0.954
Gender (M/F)	22/77	6/25	16/52	0.643
Operation time (min)	126.36 ± 13.77	132.90 ± 13.95	123.38 ± 12.71	0.001
Postoperative hospital stay (days)	4.93 ± 0.94	5.13 ± 1.15	4.84 ± 0.82	0.154
Postoperative complications	6	3	3	0.309
Location of tumor				0.827
Left thyroid gland	42	14	28	
Right thyroid gland	57	17	40	
Tumor size (mm)	6.84 ± 1.84	6.65 ± 1.89	6.93 ± 1.82	0.483
Number of LNs removed	4.79 ± 1.51	4.19 ± 1.51	5.06 ± 1.44	0.008
Removal of PTG	22 (22.2)	11 (35.5)	11 (16.2)	0.040
Positive LN metastatic	32	9	23	0.817

*LN* lymph node, *PTG* parathyroid gland

**Fig. 1:**
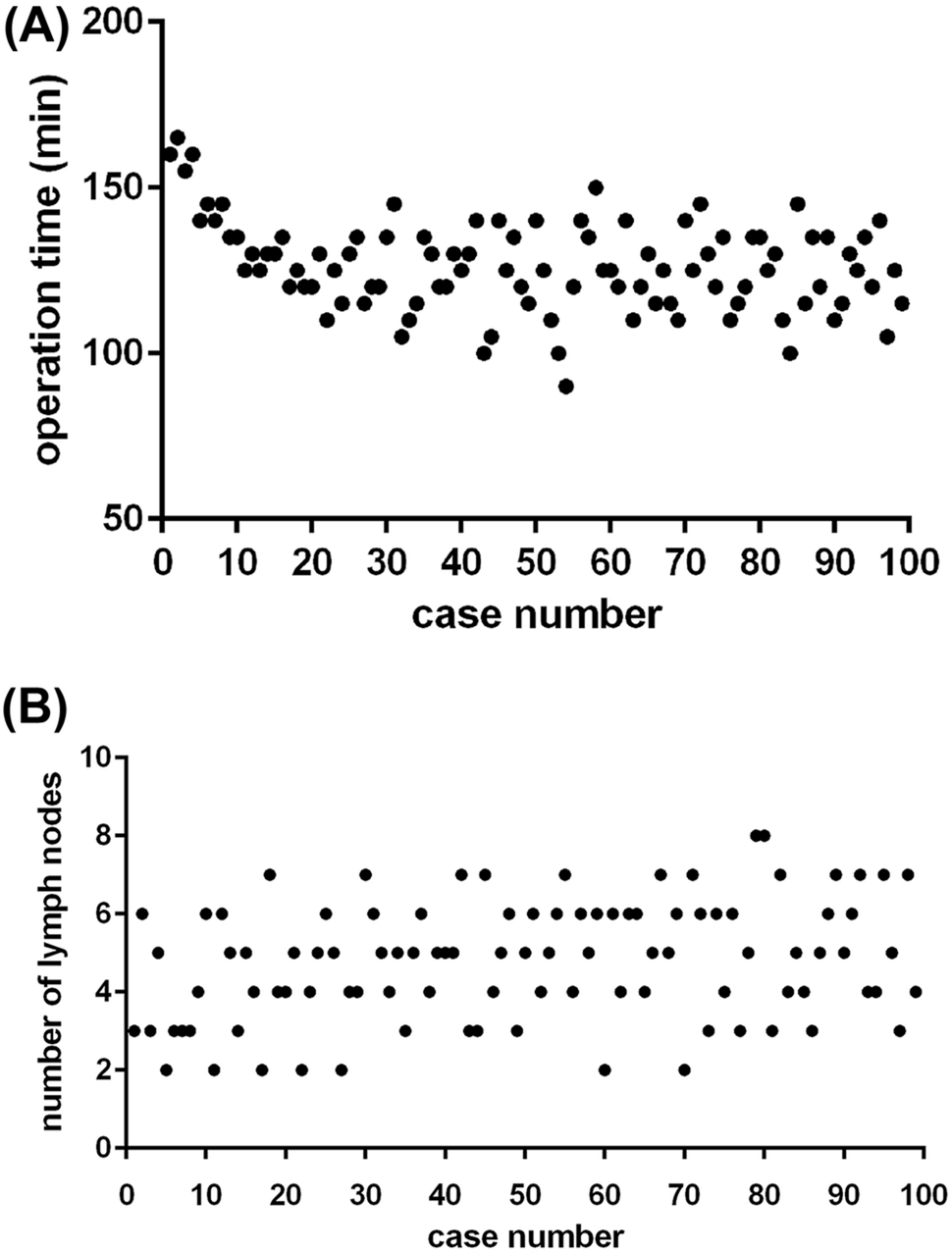
Operation time (**A**) and number of LNs removed (**B**) plotted against case number

**Fig. 2:**
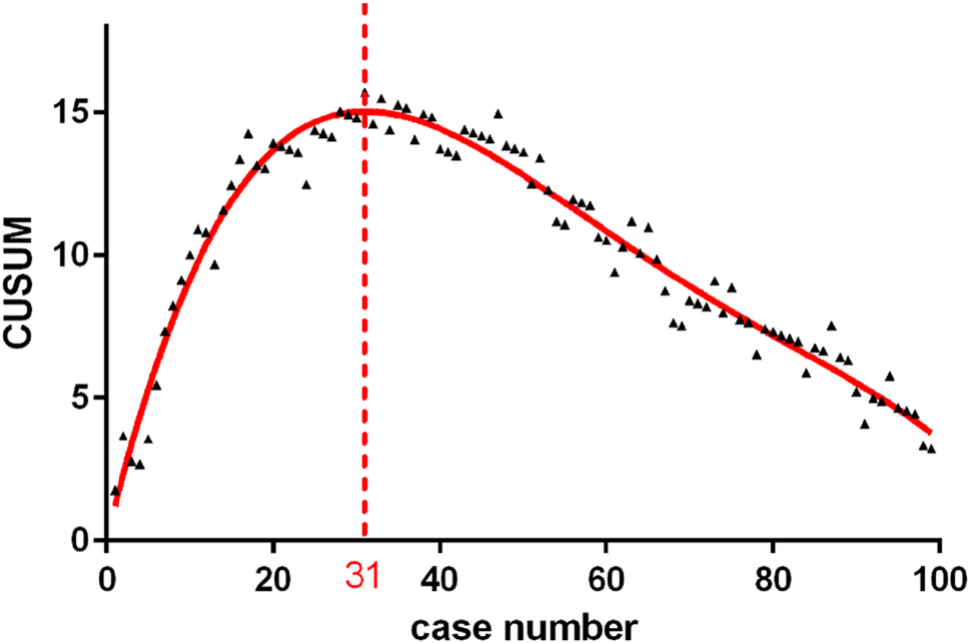
CUSUM plotted against case number

The raw data of the LNs removed and operation time are shown in chronological case order in Fig. 1. The operation time gradually decreased at about the first 20 cases and stabilized in following cases. The CUSUM learning curve is shown in Fig. 2, and the best fit for the curve was a fourth-order polynomial with equation CUSUM equaled to 4.960 × 10^−2^ × 1.196 × (Case number) − 3.153 × 10^−2^ × (Case number)^2^ + 3.091 × 10^−4^ × (Case number)^3^ − 1.099 × 10^−6^ × (Case number)^4^, which had a high *R*-value of 0.9622. The slope of the learning curve is shown in Table 2, and it turned from positive to negative when the number of cases exceeded 31. The dotted line means number 31 marked in the CUSUM graph divided the learning curve into two phases: phase 1 identified by the first 31 cases and phase 2 identified by the subsequent 68 cases. Comparisons of various parameters between the two phases identified by CUSUM analysis are presented in Table 1. Age, gender, tumor size, the location of thyroid tumor, postoperative hospital stay, and postoperative complications were similar between the two phases (*P* > 0.05) but the number of LNs removed in phase 2 was significantly more than that in phase 1 (5.06 ± 1.44 vs. 4.19 ± 1.51, *P* = 0.001). The operation time in phase 2 was 123.38 ± 12.71 min, which was shorter than that in phase 1 (132.90 ± 13.95 min), a difference that was statistically significant (*P* = 0.008). The rate of accidental removal of PTG decreased from 35.5% in phase 1 to 16.2% in phase 2 (*P* = 0.040). The detailed postoperative complications are shown in Table 3.

**Table 2:** The slope of learning curve

Case	28	29	30	31	32	33	34	35
Slope	0.0608	0.0399	0.0201	0.0013	− 0.0164	− 0.0331	− 0.0489	− 0.0489

**Table 3:** Postoperative complications

	Total (*n* = 99)	Phase 1 (*n* = 31)	Phase 2 (*n* = 68)	*P* value
Transient RLN palsy	4	2	2	0.587
Transient hypocalcemia	1	1	0	0.313
Lymphorrhagia	2	1	1	0.530
Total	6	3	3	0.374

*RLN* recurrent laryngeal nerve

## Discussion

PTMC deserves attention because of its increasing frequency among patients with papillary thyroid carcinoma in clinical practice and its implications for patient management involving young adults [1]. Conventional open thyroid surgery plays a vital role in the treatment of patients with different thyroid malignancies. However, the resultant scar after the procedure in an exposed area such as the neck is disliked by many patients, particularly so in the female population. With the development of endoscopic surgical technic, accumulation of surgical skills, and an increasing desire in postoperative quality of life, endoscopic thyroid surgery has been gradually utilized in the treatment of thyroid disease, including thyroid carcinoma. Although endoscopic thyroid surgery is controversial in thyroid carcinoma with many people speculating that endoscopic thyroid surgery may have a higher proportion of complications than conventional thyroid surgery [15, 16], a recent report has demonstrated that endoscopic thyroid surgery is an effective and safe alternative for selected patients with PTMC when compared with conventional open thyroid surgery [5].

As an advanced surgical procedure, endoscopic thyroid surgery requires skilled surgeons and can be mastered with a significant quantity of surgical experience as it requires a long learning curve. Several studies have investigated the learning curve for endoscopic thyroid surgery. Liu et al. had split their 300 first cases of endoscopic thyroid surgery into segments of 30 patients and found that the initial learning curve for endoscopic thyroid surgery required 60 cases [8]. Similarly, Pons et al. divided their first 50 cases of minimally invasive video-assisted thyroidectomy into 10-case quintiles and postulated that 10 patients represented the early stage of the learning curve [7]. These studies performed their analysis based on chronological cases split into predefined segments, with univariate analysis performed to compare means across segments. Lee et al. investigated the change in operation time and amount of drainage according to case sequence number and summarized that the endoscopic approaches reached steady state after 50 cases for the retroauricular approach and 80–90 cases for the transaxillary approach [9]. Liao et al. used the CUSUM method to evaluate the learning curve for endoscopic thyroid surgery performed by a single surgeon in their research [1]. CUSUM can be performed recursively to enable investigators to visualize the data for trends.

In previous research, the learning curve for endoscopic thyroid surgery was investigated with retrospective analysis which resulted in a variety of thyroid diseases and surgical procedures resulting in a restrained consistency of data. Before the beginning of our study, the surgeon had been proficient in endoscopic thyroidectomy but had never performed CND in endoscopic surgery. As a result, we used prospective analysis to collect connective data and investigate the learning curve for EHT with ipsilateral CND for PTMC. The previous publications limited their analysis to operation time in assessing surgical competence. Operation time is an objective assessment of technical skills. A decrease of operation time is expected with the increase in experience. However, using operation time as a sole indicator for surgical competence might not be adequate. Therefore, appropriate outcome measures are important to measure the true learning process. Thus, a multidimensional CUSUM was put into use to analyze the connective data and chart the learning curve [11].

In the present study, operation time, the number of LNs removed, the postoperative complications, and accidental removal of PTGs were used as assessment indicators for surgical competence. As the assessment indicator of technical skill, the mean operation time of these chronological cases was 126 min. This showed that the trend of operation time gradually decreased at about the first 20 cases and stabilized in subsequent cases as shown in the scatter diagram (Fig. 1). The mastery level of surgical skill for the surgeon was achieved after about 20 cases. It was much shorter than that in previous studies because the surgeon had prior experience in endoscopic thyroidectomy. However, the assessment of surgery was not only surgical skill but also the surgical outcome. We used multidimensional CUSUM method to analyze the four assessment indicators and comprehensively assess surgical competence. The CUSUM for each case was marked in the graphs and plotted on a scatter diagram. The curve-fitting method was put into use to describe the relationship between CUSUM and the case number. The best fitting for the curve was a fourth-order polynomial which had a high *R*-value of 0.9622. This high-credibility fitting curve could accurately describe the learning process. The CUSUM graph showed the learning curve ascended with the accumulation of experience at the first 31 cases and declined in the subsequent cases. It was divided into two phases, phase 1 (the first 31 cases) and phase 2 (the following 68 cases). Phase 1 represented the initial learning phase and the increase of surgical competence and phase 2 represented mastery of the procedure. There were significant differences in some parameters after contrasting phase 1 and phase 2 (Table 1). And insignificance difference in postoperative complications between two phases may be due to the limited sample sizes. The variance between phase 1 and phase 2 indicated mastery of surgery.

The optimal extent of thyroidectomy and the role of prophylactic CND for PTMC remains controversial [17, 18]. For patients with PTMC but no lymph nodal involvement, hemithyroidectomy, total or near total thyroidectomy with or without prophylactic CND is advocated [19]. In the present study, we performed EHT with prophylactic ipsilateral CND for patients. Since our surgeon had been proficient in endoscopic thyroidectomy for benign thyroid nodules, we aimed to investigate the learning curve for CND in the study. The key and difficult points for CND were ensuring sufficient dissection and avoid additional damage. Previous studies indicate that CND led to 12.1% increase in the temporary hypoparathyroidism rate with a 1.7% increased rate for permanent hypoparathyroidism and 1.3% for temporal RLN, which would impair quality of life [20]. Although there was rare hypoparathyroidism in hemithyroidectomy with CND because of bilateral PTGs, it demanded us to more carefully dissect RLN and PTGs. We traced and dissected RLN during CND in the whole course, and used an endoscopic dissector to avoid damage by collateral energy from the Harmonic Scalpel when closing to RLN. Even so, transient RLN injury developed in 3% (3/99) in these chronological cases but all recovered with 3 months postoperatively. The rate of accidental removal of PTG was 30% (30/99) although inferior and superior PTGs were carefully distinguished and dissected leading to a dramatic decline in phase 2. The only case of transient hypoparathyroidism might have occurred because the patient had congenital insufficient in the amount of PTG. We used the Harmonic Scalpel exclusively for hemostasis without any ties or clips, and no postoperative hemorrhage or hematoma occurred except for two cases of lymphatic leakage. With the accumulation of experience, the small probability of complications might be unavoidable, but the occurrence of accidental removal of PTG would reduce.

The learning curves of surgical procedure are known to vary between surgeons. This variation may be influenced by the experiences of the surgeons and assistants and the learning capacity of the surgeons. The improvements in instruments and imaging techniques also have a role in shortening the learning time. The study’s limitation is that the surgical data referred to only a single surgeon in our study. The learning curve simply provides references to other surgeons in the learning process but cannot be a criterion for judging whether the surgeons have entered the stable stage of surgical competence. To accurately judge the learning curve of ETH with prophylactic ipsilateral CND, more surgeons should be involved in future research.

In conclusion, we used of multidimensional CUSUM method to analyze the 99 chronological cases, and the learning curve was depicted according to the CUSUM graph. The study results suggested that the surgeon experienced in endoscopic thyroidectomy may become proficient in EHT with prophylactic ipsilateral CND after 31 cases, and the safety and feasibility of our endoscopic surgery can also be demonstrated even in the initial part of the learning curve.
